# Mutation Detection by Real-Time PCR: A Simple, Robust and Highly Selective Method

**DOI:** 10.1371/journal.pone.0004584

**Published:** 2009-02-25

**Authors:** John Morlan, Joffre Baker, Dominick Sinicropi

**Affiliations:** Genomic Health, Inc., Redwood City, California, United States of America; Stanford University, United States of America

## Abstract

**Background:**

Molecular tests for diagnosis of disease, particularly cancer, are gaining increased acceptance by physicians and their patients for disease prognosis and selection of treatment options. Gene expression profiles and genetic mutations are key parameters used for the molecular characterization of tumors. A variety of methods exist for mutation analysis but the development of assays with high selectivity tends to require a process of trial and error, and few are compatible with real-time PCR. We sought to develop a real-time PCR-based mutation assay methodology that successfully addresses these issues.

**Methodology/Principal Findings:**

The method we describe is based on the widely used TaqMan® real-time PCR technology, and combines Allele-Specific PCR with a Blocking reagent (ASB-PCR) to suppress amplification of the wildype allele. ASB-PCR can be used for detection of germ line or somatic mutations in either DNA or RNA extracted from any type of tissue, including formalin-fixed paraffin-embedded tumor specimens. A set of reagent design rules was developed enabling sensitive and selective detection of single point substitutions, insertions, or deletions against a background of wild-type allele in thousand-fold or greater excess.

**Conclusions/Significance:**

ASB-PCR is a simple and robust method for assaying single nucleotide mutations and polymorphisms within the widely used TaqMan® protocol for real time RT-PCR. The ASB-PCR design rules consistently produce highly selective mutation assays while obviating the need for redesign and optimization of the assay reagents. The method is compatible with formalin-fixed tissue and simultaneous analysis of gene expression by RT-PCR on the same plate. No proprietary reagents other than those for TaqMan chemistry are required, so the method can be performed in any research laboratory with real-time PCR capability.

## Introduction

It is widely accepted that cancer is a genetic disease caused by the accumulation of mutations and chromosomal aberrations [1]. Mutations in oncogenes and tumor suppressor genes determine the phenotype of a tumor: its location, aggressiveness, and sensitivity to therapeutics. Germ line mutations may predispose to risk of developing cancer and influence the host response to the tumor. The pathological features of virtually all tumors are driven by acquisition of somatic (within tumor) mutations that alter processes controlling cellular proliferation, mobility, and apoptosis [2]. Detection of either germ line or somatic mutations has the potential to influence decision-making in oncology. Increasingly, somatic mutations have been proposed as biomarkers for cancer prognosis and prediction of therapeutic efficacy. Recent examples include the prediction of response [3]–[5] or resistance to certain oncology drugs based on mutations in EGFR [6] or Kras [7]–[10]. This report describes a PCR-based assay that is well-suited for the detection of either germ line or somatic mutations at a known base position such as those that occur in Kras and BRAF.

Due to the cellular heterogeneity of most solid tumors, somatic mutations in a gene can be present in low abundance within a very high background of wild type sequence that may only differ from mutant at a single nucleotide. Thus, detection of somatic mutations poses a greater analytical challenge than detection of germ line mutations. In this report we have adopted the terminology of Liu and Sommer [11] for the analytical characterization of mutation assays. *Sensitivity* is defined as the minimum amount (number of copies or mass) of a template that can be detected. The *specificity* of a mutation assay is the maximum amount of a mismatched template that is undetectable and *selectivity* is the relative assay response to the matched and unmatched template. Selectivity is often expressed as a ratio or percentage. For example, an assay that can detect 1 mutant template in the presence of 100 wild type templates is said to have a selectivity of 1∶100 or 1%. Genotyping assays only need to have a selectivity of 50%, that is, the assays must be able to detect 1 mutant template in the presence of one wild type template. However, selectivity greater than 1∶1000 might be required for detection of clinically significant somatic mutations, for example, when monitoring blood for early detection of cancer, monitoring disease progression, and response to therapy [12], [13].

Several methods exist for detection of somatic mutations by real-time PCR. These methods include use of allele-specific competitive blocker PCR [14], blocker–PCR [15], [16], real-time genotyping with locked nucleic acids [17], [18], restriction enzymes in conjunction with real-time PCR [19], and allele-specific kinetic PCR in conjunction with modified polymerases [20]. Additional methods include ARMS-PCR [21], TaqMAMA [22],and FLAG-PCR [23]. These methods require either the use of modified bases, special enzymes, or additional proprietary reagents or procedures. We wished to develop a simple, robust, highly sensitive, and selective method that is compatible with standard processes used for gene expression analysis by real-time RT-PCR [24].

A widely used strategy for detecting DNA sequence variants is allele-specific PCR in which one or both primers are designed to anneal at sites of sequence variation [25]. Ideally, a primer whose sequence matches a specific variant should selectively amplify only that variant; however, in practice, significant mismatched amplification typically occurs. It is common practice to anchor the 3′ end of the allele-specific primer at the mutant base in order to selectively amplify the mutant template. This strategy reduces but does not eliminate amplification of the wild-type allele. The amount of this non-specific amplification has been found to vary widely depending on the particular base mismatch between the allele-specific primer and the wild-type sequence [18], [26], [27]. The variability of non-specific amplification typically requires a process of trial and error when designing highly selective mutation assays [18], [21], [22]. The assay method reported here utilizes a combination of allele-specific PCR primers, a blocker oligonucleotide to suppress amplification of the wild type allele, and a set of reagent design rules that consistently produce highly selective assays for a wide variety of single point substitutions, insertions, or deletions. We refer to the modified assay by the acronym ASB-PCR (Allele-Specific Blocker PCR). Features of the method include the ability to detect mutations in either DNA or RNA with a high level of sensitivity and selectivity. No proprietary reagents are required so the method can be performed in any laboratory with real-time PCR capability. Moreover, the assay is compatible with the process stream for real-time RT-PCR, enabling mutation analysis to be carried out alongside gene expression analysis of the same clinical specimen.

Mutations in Kras were chosen for the initial development and characterization of the RT-PCR assay method based on their clinical importance and high frequency in colorectal cancer. Kras mutations are found in approximately 32% of colorectal tumors, with eight single point substitutions accounting for the majority of the mutations [28]. Kras mutations predict profound tumor resistance to drugs that target the epidermal growth factor receptor [7]–[10] and have also been associated with tumor stage and risk of recurrence [28], [29].

## Materials and Methods

### Sources of Nucleic Acids

Cell lines of known Kras genotype were obtained from ATCC (Manassas, VA) (SW480, DLD-1, A549, MIA Paca-2, SW1116, Colo 320) or the European Collection of Cell Cultures (Wiltshire, UK) (LS174T). RNA was extracted directly from frozen cell pellets using an RNEasy kit® (QIAGEN, Valencia, CA) and quantitated by A_260_. Colorectal cancer tumor RNA and DNA were extracted from serial sections (3×10 µm sections per extraction) of eighty-two formalin-fixed paraffin-embedded (FPE) tissues obtained from ProteoGenex (Culver City, CA) using an Epicentre MasterPure™ kit (Madison, WI) according to manufacturer's instructions. RNAs were quantitated by the RiboGreen Assay® and DNAs by the PicoGreen Assay® (Invitrogen, Carlsbad, CA). Genomic DNA from HeLa cells was purchased from BioChain (Hayward, CA). HeLa cell RNA was purchased from Applied Biosystems/Ambion (Austin, TX).

Synthetic RNA templates for selected mutations were prepared using a method for generating long templates from shorter oligonucleotides [30]: Two synthetic oligonucleotides were designed to be partially complementary at their 3′ ends and have a combined length that encompassed the mutation assay amplicon. The oligonucleotides were denatured at 95°C for three minutes and then cooled rapidly on ice. The products were extended in a Klenow reaction containing 25 pmol annealed oligonucleotidess, 5 Units Klenow Fragment (New England Biolabs, Ipswich, MA), 1 mM dNTPs and 1× NEB2 Buffer (New England Biolabs). A MegaShortscript™ IVT reaction (Applied Biosystems/Ambion) was performed in 20 µL according to manufacturer's instructions with one µL of a 1∶5 dilution of Klenow reaction. The IVT reaction was run at 37°C overnight followed by treatment with 1 µL DNAseI at 37°C for 15 minutes. Reactions were purified with an RNEasy® Kit (QIAGEN) and IVT yield was determined by A_260_ and confirmed by limiting dilution TaqMan® assay [31]. A dilute synthetic DNA oligonucleotide carrying the mutation G215C (Assay Mut6, see Table 1) was obtained from Eurogentec North America (San Diego, CA). Concentration was verified by limiting dilution TaqMan® assay. Oligonucleotide primers and probes were obtained from Integrated DNA Technologies (Coralville, IA).

**Table 1:** Kras mutations and their frequency in Colon Cancer.

Assay Name	Nucleotide Substitution	Amino Acid Change	Percent of Kras mutations in Colon Cancer^a^	Percent of Colon tumors^b^	Percent of tumors in this study^c^
Mut1	G216T	G12V	22.7%	7.3%	13.4%
Mut2	G216A	G12D	32.2%	10.3%	9.8%
Mut3	G219A	G13D	21.6%	6.9%	9.8%
Mut4	G215A	G12S	8.2%	2.6%	3.7%
Mut5	G215T	G12C	9.9%	3.2%	4.9%
Mut6	G215C	G12R	0.9%	0.3%	0%
Mut7	G216C	G12A	4.0%	1.3%	0%
Total			99.6%	31.9%	41.5%

a Data derived from Samowitz et al. [28]. Table values represent the frequency of the specified mutation as a percentage of total observed Kras mutations. The total is not 100% because Samowitz et al. reported an additional mutation at a frequency of 0.4%.

b The frequency of the specified mutation calculated as a percentage of total tumor specimens tested by Samowitz et al. [28]. Four of 449 tumors had two mutations.

c Determined in DNA extracted from 82 formalin-fixed paraffin-embedded colorectal cancer specimens.

### TaqMan® RT-PCR Assays

Reverse transcription was performed using an OmniScript RT Kit (QIAGEN) according to the manufacturer's instructions in a 10 µL volume with 50 nM of each reverse primer. TaqMan PCR was performed with an RT volume of up to 1.25 µL in a 5 µL assay with 1× TaqMan Universal PCR Master Mix (no UNG)™ (Applied Biosystems, Foster City, CA), 900 nM primers, 200 nM probe and 3600 nM blocker. One ng of RNA or 0.4 ng of DNA extracted from FPET were analyzed in each PCR reaction, unless noted otherwise. Standard TaqMan thermocycling conditions were used: 10 min. at 95°C, 40 cycles of 20 sec. at 95°C, 45 sec. at 60°C. All PCR assays were run in triplicate or at higher replication when deemed necessary.

A list of the oligonucleotides used for all of the PCR mutation assays is provided in Table S1. Assays have been numbered for ease of reference in the text. Assays that use the forward primer as the discriminating primer are appended with “.1” and assays that use the reverse primer in this fashion are appended with “.2”. PrimerExpress™ (Applied Biosystems, Foster City, CA) was used for assay design and estimation of oligonucleotide melting temperature.

### Sequence Analysis

Bi-directional sequencing (one pass per direction) was performed by SeqWright (Houston, TX) on 53 ProteoGenex FPE tissue gDNA samples using dye-terminator chemistry (ABI BigDye® v3.1) on an ABI 3730xl DNA sequencer. Base calls were determined manually at SeqWright according to the following rules: Minimum phred scores of 20 were required to call bases. Traces were aligned to a reference sequence and identical results were required in both forward and reverse sequencing strands to make minor base calls. In the case where only one sequence trace was available minor alleles were called if the secondary peak was greater than 20% of the primary peak In all cases calling a minor allele as present was weighed against the amount of noise in the immediate vicinity of the peak of interest. SeqWright had no prior knowledge of sample genotypes as determined by our assays.

## Results

### Description of the Assay Method

**Figure 1:**
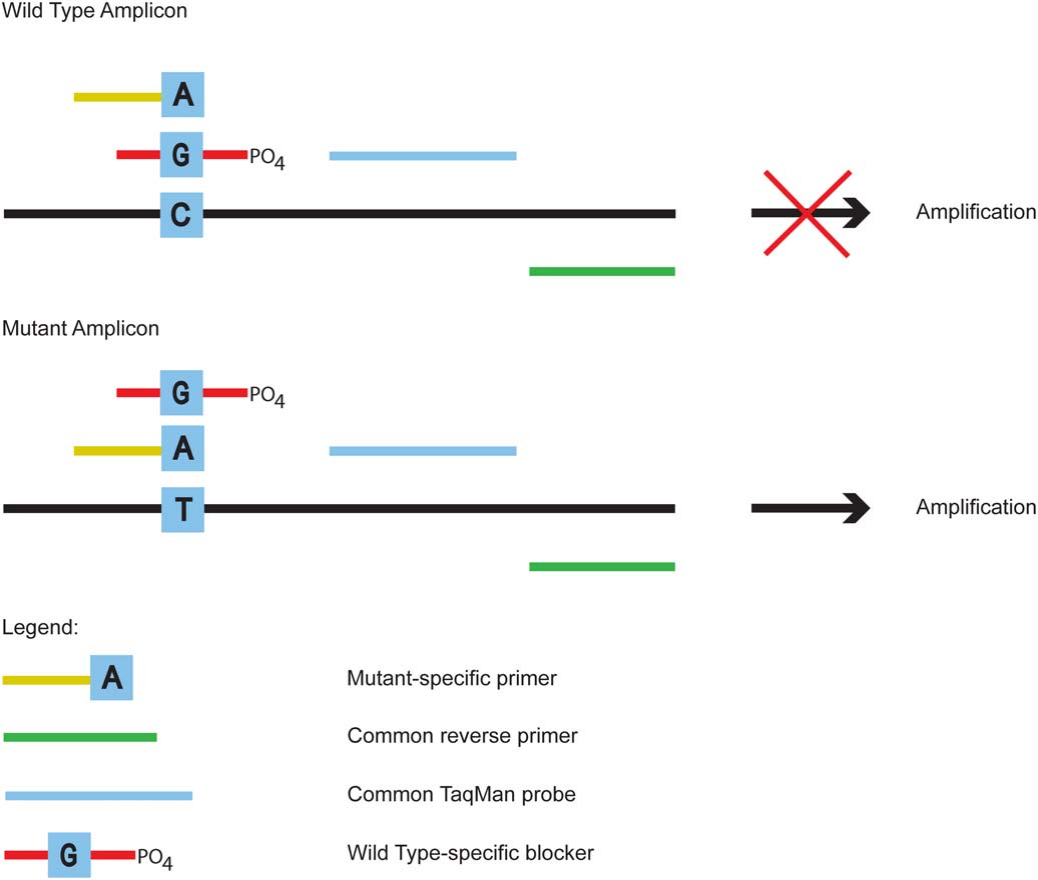
Diagram to illustrate the assay method.

We sought to develop a set of assay design rules that would improve and standardize the performance of allele-specific PCR so that amplification of primer∶template mismatches would generally be strongly suppressed. To accomplish this, two criteria were introduced into our assay designs. First the mutant-specific primer (Figure 1) was shortened at its 5′-end to reduce its T_m_ to approximately 10°C below the anneal/extend temperature of the assay. Second, a blocking oligonucleotide, complementary to the wild type sequence but phosphorylated at the 3′-end to prevent extension, was added to further suppress nonspecific amplification of the wild type allele by the mutant-specific primer. The blocking reagent was designed to have the variant base position approximately in the middle of the oligonucleotide and to partially overlap the sequence of the mutant-specific primer. Two additional reagents, a second PCR primer and a Taqman Probe, are needed for real-time PCR of either the wild type or variant template (although other detection systems such as SYBR green or molecular beacons should also work).

### Allele-Specific Primer Development with synthetic RNA templates

**Figure 2:**
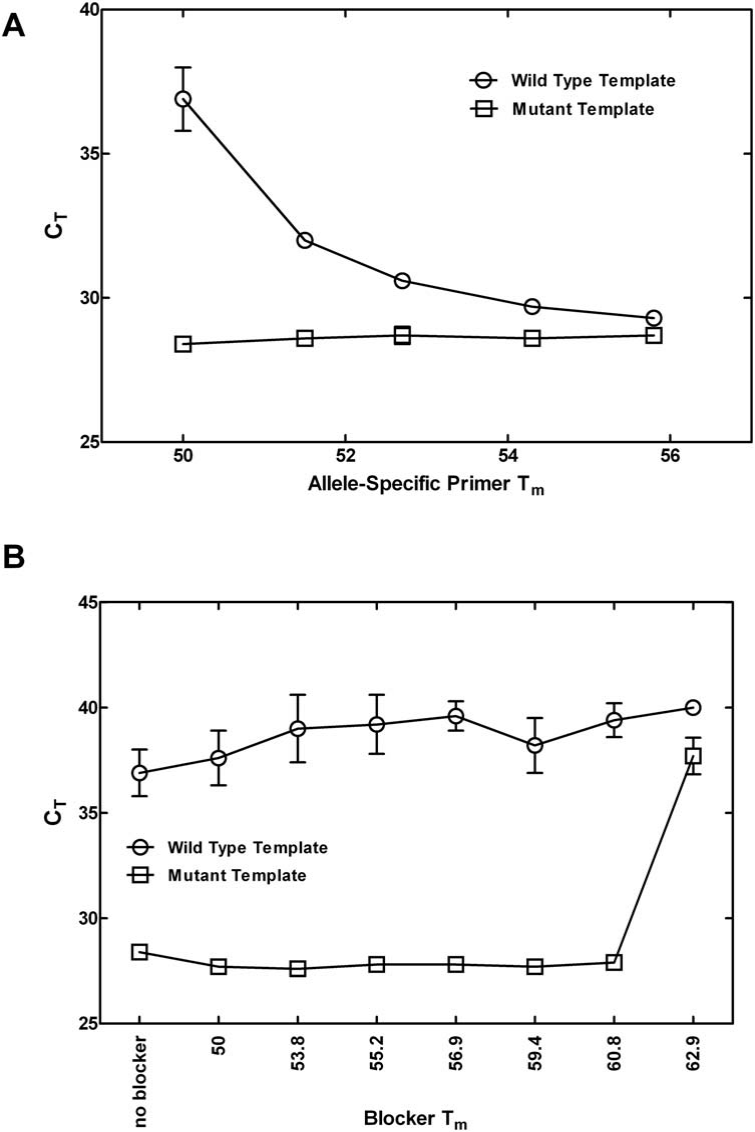
Optimization of allele-specific primer and blocker Tm. C_T_ values are for the Kras Mut1.1 assay (G216T). A mutant-specific primer was used to assay either a wild type or mutant RNA template. Circles represent response to wild type synthetic RNA. Squares represent response to mutant synthetic RNA. Error bars represent 95% confidence intervals. The final version of this assay included a blocker oligonucleotide with a T_m_ of 60.8°C and a variant-specific primer with a T_m_ of 50°C. A. The effect of primer T_m_ on variant-specific assay C_T_. B. The effect of blocker oligonucleotide T_m_ on variant-specific assay C_T_.

In a published study of 1413 colon tumors [28] eight substitutions accounted for 100% of the mutations found in Kras. Our initial assay development experiments were carried out on the three most commonly occurring mutations in Kras, G216T, G216A and G219A (Table 1) represented by assays Mut1.1, Mut2.1 and Mut3.1, respectively, all of which use the forward primer as the discriminating primer. The effect of primer T_m_ and concentration were investigated for each of the assays using cDNA derived from synthetic RNA templates. Reduction in concentration of the discriminating primer from the standard 900 nM down to as low as 28 nM did not improve assay selectivity and sometimes had deleterious effects on sensitivity (data not shown). The T_m_ of the allele-specific primer was varied by shortening the 5′-end while keeping the 3′-end anchored on the variant base. For each assay, eight allele-specific primers of differing lengths, ranging in T_m_ from approximately 50°C to 60°C, were tested using synthetic wild type or mutant RNA templates. PCR cycling conditions were kept constant at the manufacturer's recommendation for TaqMan® real-time RT-PCR assays (see Methods; the recommended anneal/extend temperature of 60°C was maintained throughout these experiments). Results obtained with assay Mut1.1 using allele-specific primers with T_m_ values between 50°C and 56°C are summarized graphically in Figure 2A. When assay Mut1.1 was directed against a wild-type RNA template, producing a 3′ end allele-specific primer∶template mismatch of T∶C, C_T_ values increased as a function of decreasing discriminating primer T_m_. C_T_ values were unchanged, however, when the same primers were used to assay the mutant target (3′ end primer∶template match of T∶A). The difference in C_T_ value (ΔC_T_) between the wild type and mutant templates was largest at a primer T_m_ of approximately 50°C. Very similar results were obtained when allele-specific primer T_m_ was varied in the Mut2.1 and Mut3.1 assays (data not shown). The maximum ΔC_T_ for each mutation assay was obtained with a primer T_m_ of approximately 50°C, which produced ΔC_T_ values equal to 8.4, 11.5, and 10.0 for Mut1.1, Mut2.1, and Mut3.1, respectively. Because it is difficult to predict the T_m_ at which the sensitivity of an assay will be adversely affected, all allele-specific primers were designed to have a T_m_ of approximately 50°C, which is 10°C below the anneal/extend temperature of the standard cycling conditions. The effect of lower primer T_m_, below 50°C, was investigated in a separate experiment using wild type and mutant templates extracted from cell lines. At primer melting temperatures below 50°C the ΔC_T_ was sometimes unchanged but frequently decreased due to a loss in sensitivity for detection of the mutant templates (Figure S1).

### Blocker Development with synthetic RNA templates

Because wild type allele abundance may occur in great excess over the mutant target of interest in tumors we took further measures to improve the assay selectivity. Blocking oligonucleotides were synthesized that are both complementary to the wild type allele at the position of the variant base and phosphorylated at their 3′ ends to prevent polymerase extension. These blocking oligonucleotides were evaluated for their effect on selectivity using synthetic wild type or mutant RNA templates in the Mut1.1, Mut2.1, and Mut3.1 assays. Blockers were evaluated with respect to concentration, length/Tm and position of the mutation in the oligonucleotide. Blockers with the variant base position located approximately in the middle of the oligonucleotide were found to produce the best assay selectivity (data not shown).

We studied the effect of blocker T_m_, ranging from approximately 50°C to 65°C, on cross-amplification of a wild type template by a mutant-specific primer. A T_m_-dependent inhibition of wild type template amplification was observed. An example of this effect is shown in Figure 2B for assay Mut1.1 using a 50°C allele-specific primer and seven different blocking oligonucleotides. Assay selectivity was improved by up to 3.7 C_T_ units by the use of blockers with T_m_ greater than or equal to approximately 57°C. The slight dip observed with the 59.4°C blocker was the only anomaly and likely was the result of high variability associated with high C_T_ values. Decreased sensitivity for detection of the mutant template was only seen when the blocker T_m_ was 2°C or more higher than the PCR anneal/extend temperature (60°). Similar effects of blocker T_m_ on amplification of mutant and wild type templates were observed for the Mut2.1 and Mut3.1 assays (data not shown). For all three mutation assays, the combination of a blocker with T_m_ of at least 54°C and allele-specific primer with T_m_ of approximately 50°C produced the greatest selectivity without measurable losses in sensitivity.

We checked the variability of wild type template inhibition as a function of blocker concentration for the Mut1.1, Mut2.1 and Mut3.1 assays. An increasing dose-dependent inhibition of amplification was observed up to approximately 3.6 µM blocker concentration and reached a plateau at about 8 C_T_. Wild type-specific blockers had no effect on mutant synthetic RNA amplification at concentrations as high as 14.4 µM (data not shown). Blockers designed with the variant base position approximately in the middle of the oligonucleotide and a T_m_ approximately equal to the anneal/extension temperature improved the selectivity of ASB-PCR assays for those Kras mutations that had a ΔC_T_ less than ∼11 using a low T_m_ primer alone (Table 2).

**Table 2:** Effect of Reagent Design on Mutation Assay Performance in Cell Line RNA or DNA.

Assay Name b	Primer∶Template mismatch	Allele-specific primer only c	+Blocker d	+Low T_m_ primer e	+Both f
		ΔC_T_ a			
Mut1.1	T∶C	1.3	8	10.4	15.6
Mut1.2	A∶G	8.3	14.1	15.3	15.5
Mut2.1	A∶C	2.3	8.4	11.5	13.3
Mut2.2	T∶G	1.4	5.5	8.1	11.6
Mut3.1	A∶C	0.8	2.7	9.9	13.3
Mut3.2	T∶G	−0.3	−0.3	4.6	7.5
Mut4.2	T∶G	−0.7	0.4	5.5	9.1
Mut5.1	A∶G	1.8	7	7.9	13.8
Mut6.1^g^	G∶G	11.5	14.4	16.3	16.1
Mut7.1	G∶G	10.3	12.8	15.7	15.4

a ΔC_T_ is the difference in C_T_ obtained from wild type and mutant templates in the allele-specific mutation assay.

b Assay names ending in “.1” were designed with the forward primer to be specific for the mutant sequence and assay names ending in “.2” were designed with the reverse primer specific for the mutant sequence.

c ΔC_T_ obtained when an allele-specific primer with T_m_ about 60° was used without a blocker.

d ΔC_T_ obtained when a blocker was used in combination with an allele-specific primer.

e ΔC_T_ obtained when a low T_m_ allele-specific primer was used.

f ΔC_T_ obtained when both a blocker and low T_m_ allele-specific primer were used in combination.

g Synthetic mutant DNA was used for Assay Mut6.1 due to the unavailability of cell lines carrying this mutation. Wild type DNA was obtained from HeLa cells.

The value of including a blocker in eleven ASB-PCR assays was examined using wild type and mutant templates extracted from cell lines (Figure S1). The presence of a blocker improved assay selectivity for eight of the assays by increasing the C_T_ for detection of the wild type template. For the three assays where inclusion of a blocker did not improve assay selectivity no deleterious effect on the assay was observed. Therefore, we chose to include blockers in the standardized assay design rules described below.

### Standardized Assay Design Rules

Standardized design rules (Table 3) were developed for ASB-PCR assays based on the tests with synthetic RNAs described above. The goal of these rules was to design assays with selectivity of 1∶1000 or more on the first design without a need for additional optimization of the assays. The rules call for 1) an allele-specific primer that is 10°C below the anneal/extend temperature in the cycling conditions, 2) a non-extendable blocker, specific for the wild type sequence, with the variant base position approximately in the middle of the oligonucleotide, 3) a blocker T_m_ approximately equal to but not greater than the cycling anneal/extend temperature, and 4) a blocker concentration that is 4-fold greater than that of the allele-specific primer. In addition, blocker oligonucleotides that are developed for use in a TaqMan® -based system may overlap with the fluorescent probe by a few bases. These rules were used to create several ASB-PCR assays targeting Kras mutations G216T, G216A, and G219A using the reverse primer as the allele-specific primer (assays Mut1.2, Mut2.2 and Mut3.2, respectively). In addition, we chose to develop assays targeting four Kras mutations (Mut4-7, Table 2) based on their frequency in solid tumors and association with resistance to anti-EGFR therapeutics [8], [28], [29].

**Table 3:** ASB-PCR Design Rules.

Reagent	Properties
Allele-Specific Primer	1. either the forward or reverse primer
	2. 3′-end is anchored on the variant base
	3. T_m_ is 10°C below PCR extension temperature
Blocker	1. Designed to same strand as the allele-specific primer
	2. Discriminating base is approximately in the middle of the oligonucleotide
	3. Complementary to the wild type sequence
	4. Not extendable by polymerases (phosphorylated on 3′-end)
	5. T_m_ is approximately equal to, but not greater than, the PCR extension temperature
	6. Used at 4× the concentration of the allele-specific primer

### Assay Performance in Cell Line RNA or DNA

All of the Kras mutation assays were tested with and without the blocker and allele-specific primer modifications as specified in the design rules to determine the effects these modifications have on assay selectivity. For Assays Mut1-5 and Mut7 these experiments were conducted using RNA extracted from cell lines of known Kras genotype. Cell lines carrying the G215C (Mut6) mutation were unavailable; the performance of Assay Mut6 was evaluated using synthetic mutant DNA in conjunction with wild type DNA obtained from HeLa cells. The results are summarized in Table 2. The difference in C_T_ (ΔC_T_) between wild type and mutant template C_T_ values which serves as an approximation of assay selectivity was increased when either blockers or low T_m_ discriminating primers were introduced to the assays. In most cases the magnitudes of these effects were approximately additive; the largest increases in ΔC_T_ occurred when these modifications were used in combination. In the three cases where the effects did not appear to be additive, Mut1.2, Mut6.1 and Mut7.1, the un-modified versions of the assays (no blockers or low T_m_ primers) already exhibit a high degree of selectivity.

Assay selectivity was estimated by serially diluting RNA extracted from mutant cell lines into RNA extracted from wild type cell lines (Table 4). The mass of wild type RNA was divided by the mass of mutant RNA to calculate an approximate ratio of wild type to mutant alleles at each dilution. The selectivity of each assay was estimated by determining the intersection of the upper 95% confidence interval of the regression of observed C_T_ response on mutant RNA serial dilution with the lower 95% confidence limit of the observed C_T_ response of a wild type-only RNA control. The amount of mutant RNA was estimated by interpolation from the best-fit line at this intersection. The total amount of RNA assayed was divided by the interpolated amount of mutant RNA to determine the selectivity of the assay. In all cases the assays had selectivities of 1∶1000 or greater. Mutant RNA inputs between 2 to 250 pg could be discriminated from a thousand-fold or greater excess of wild type RNA, depending on the assay tested. (These estimates of assay selectivity were conservative because most of the mutant RNA cell lines used in this mixture study were heterozygous for mutant alleles and as such had approximately half as many mutant alleles per unit mass of total RNA as compared with a homozygous mutant The one exception was cell line SW480 which was homozygous for the G216T mutation. The effect of heterozygosity was not accounted for in the calculations summarized in Table 4.)

**Table 4:** Performance of Mutation Assays using RNA or DNA Extracted from Cell Lines.

Assay Name	Cell Line Mixture (Mutant/Wild Type)	Mutation Assay Result: Mixed Cell Lines		Mutation Assay Result: Wild type Cell Line		ΔC_T_	Selectivity
		C_T_	SD	C_T_	SD		
Mut1.2^a^	SW480/COLO 320	25.8	0.45	36.7	0.38	10.9	1,600
Mut2.1^a^	LS174T/HeLa	26.1	0.27	38.6	1.22	12.5	1,000
Mut3.1^a^	DLD-1/HeLa	23.7	0.16	37.7	1.84	14	1,000
Mut4.2^a^	A-549/HeLa	24.6	0.25	36.3	0.88	11.7	1,500
Mut5.1^a^	MIA PaCa-2/HeLa	25.1	0.37	39.6	0.68	14.5	9,000
Mut6.1^b^	synthetic/HeLa DNA	23.6	0.04	40	0	16.4	15,625
Mut7.1^a^	SW1116/HeLa	23.1	0.62	40	0	16.9	32,000

a The C_T_ and standard deviation (SD) comparing a 50/50 wild type/mutant cell line mixture (32 ng each) with wild type alone (32 ng) using the indicated assay. Wild type cell lines used were either COLO 320 or HeLa, depending on availability at the time. Cell line SW480 is homozygous for Mut1 (G216T); all other cell lines are heterozygous for the indicated mutations. Linearity of all assays ranged from 0.992–0.999. Efficiency for all assays ranged from 92%–116%. ΔC_T_ is the difference between the C_T_ obtained from Wild Type Cell Line C_T_ – Mixed Cell Line C_T_. Selectivity was measured as described in Results.

b The C_T_ and standard deviation (SD) comparing a 50/50 wild type cell line DNA/mutant synthetic DNA (15,625 copies each) mixture with wild type DNA alone (15,625 copies) using the Mut6.1 assay. Synthetic mutant DNA was used for Assay Mut6.1 as no cell line carrying this mutation was available. Limiting Dilution Assay analysis was used to determine the number of copies of synthetic template as well as the number of wild type Kras alleles in HeLa DNA.

**Figure 3:**
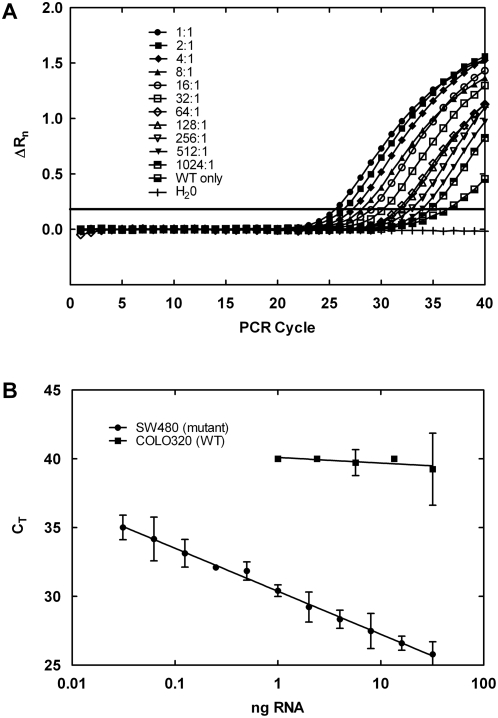
Sensitivity and selectivity of the Mut1.2 assay. A. Detection of cell line RNA containing Kras G216T mutant diluted into wild type cell line RNA using the Kras Mut1.2 assay. ΔR_n_ is the difference between the normalized fluorescence of the TaqMan reporter probe at each PCR cycle and the background fluorescence measured during the first 15 PCR cycles. Each curve represents the time course of PCR assays (average of triplicate measurements) at each dilution. The horizontal line at ΔR_n_ = 0.2 represents the threshold for determination of C_T_ for the individual amplification curves. B. Serial-dilutions of RNA extracted from wild type COLO320 (filled squares) and mutant SW480 (filled circles) cell lines submitted to the Kras Mut1.2 assay. Error bars represent 2 times the standard deviation of triplicate determinations.

Amplification plots of the real-time background-corrected fluorescence versus PCR cycle number for assay Mut1.2 are shown in Figure 3A. A dose-dependent shift of the amplification curves to the right was observed with increasing dilution of the sample. In our laboratory, the C_T_ is defined as the PCR cycle number at which the amplification curve exceeds a background-corrected fluorescence of 0.2. These data illustrate that the amplification curve for the 1∶1024 dilution was easily resolved from the sample containing only wild type RNA.

The selectivity of the mutation assays was also indicated by the relative dose-response characteristics of mutant and wild type RNA. An example, shown in Figure 3B, is the titration of mutant (SW480) and wild type (COLO 320) RNA in the Mut1.2 (G216T) mutation assay. Even the highest concentration of wild type RNA tested did not produce a C_T_ that was significantly different from 40.

### Assay Performance in Formalin-Fixed Tumor Specimens

We sought to assess the performance of ASB-PCR assays with both blockers and low T_m_ primers by genotyping DNA from formalin-fixed paraffin-embedded (FPE) tissue specimens. FPE specimens typically contain fragmented and chemically modified nucleic acid [24]. Seven Kras mutations were evaluated in DNA extracted from 82 FPE colorectal tumor specimens of unknown Kras status. Mutation status assignments were made by comparing mutant C_T_ assay values in the FPE samples to a classification line obtained using serial dilutions of wild type HeLa cell DNA. The classification line was derived as the lower bound of the 95% prediction interval from a regression model estimated using data from the HeLa wild type control. Samples that produced mutant assay C_T_ values below the classification line were called present for that mutation. The frequency of mutations detected by the ASB-PCR assay (Table 1) was similar to data reported by other investigators using different methods [28]. Sufficient DNA was available from 53 of the 82 samples for confirmatory DNA sequencing. The quality of DNA from 44 of the 53 samples was sufficient for sequencing. Mutation calls by ASB-PCR were highly concordant with sequence analysis with a statistical sensitivity and selectivity of 100% and 92.6%, respectively. Allele calls obtained by ASB-PCR for each of the individual mutation assays and by sequence analysis are summarized in Table S2.

**Figure 4:**
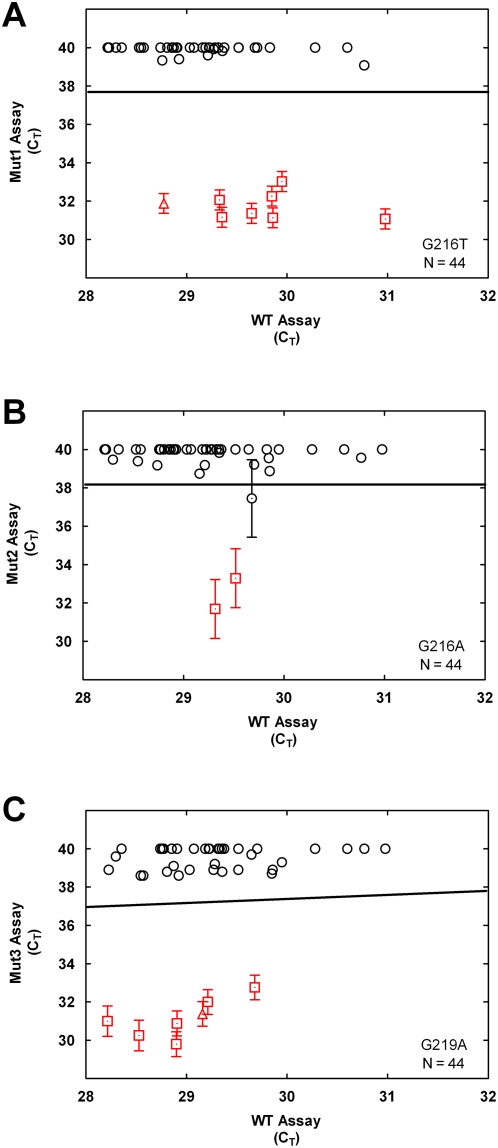
Comparison of mutation detection by ASB-PCR and nucleic acid sequencing. ASB-PCR results are shown for the 44 randomly-selected FFPE colorectal tumor specimens for which sequencing data were available. Genomic DNA extracted from 44 randomly-selected FFPE colorectal tumor specimens was submitted to ASB-PCR assays A) Kras G216T, assay Mut1.1, B) Kras G216A, assay Mut2.1, and C) Kras G219A, assay Mut3.1. In each graph the C_T_ values measured in the wild type Kras assay (x-axis) is plotted vs. the C_T_ values measured in the specified Kras variant allele assay (y-axis). Samples were assayed at 0.4 ng of DNA per well. The solid line represents the classification boundary, which was derived as the lower 95% prediction limit of a linear regression of variant-specific assay C_T_ response on a titration of wild type samples submitted to the variant-specific assay. Error bars represent 95% confidence limits based on a pooled estimate of standard error for all samples with a mean C_T_ less than 35. Note that samples for which the 95% confidence intervals overlapped were designated as wild type. Circles: (Ο) Samples called wild type by both PCR and sequencing; Squares (□): samples called mutant by both PCR and sequencing; Triangles (▵): samples called mutant by PCR but wild type by sequencing.

Two samples that typed positive by ASB-PCR, for substitutions G216T and G219A, were negative by sequence analysis (Table S2). Several lines of evidence suggest that these discordant samples did, in fact, harbor Kras mutations. First, the C_T_ values obtained for these samples in the Mut1 and Mut3 assays clearly distinguished them from wild type specimens. The C_T_ values from ASB-PCR analysis of all 44 samples are depicted graphically in Figures 4 A–C. The C_T_ values from assays with the mutant alleles are on the Y-axes and those from assays with the wild type allele are on the X-axes. Both discordant samples (triangles in Figures 4A and 4C) cluster with other mutation positive samples and are well-separated from the classification line for the wild type population. Second, the mutation calls for these samples made from the sequencing electropherograms were complicated by experimental noise that may have resulted from poor sample quality (data not shown). In addition, for one of the discordant samples sequencing data was only obtained from one strand of the sequencing template. Inspection of the electropherograms for these samples in fact revealed minor peaks that are consistent with the presence of the mutations called by ASB-PCR. Third, as described below, ASB-PCR analysis of RNA extracted from the same samples confirmed the mutation calls made by ASB-PCR analysis of DNA. Taken together, these results support the view that the two discordant samples were true positives for Kras mutations that were undetected by sequence analysis.

### ASB-PCR analysis of RNA

**Figure 5:**
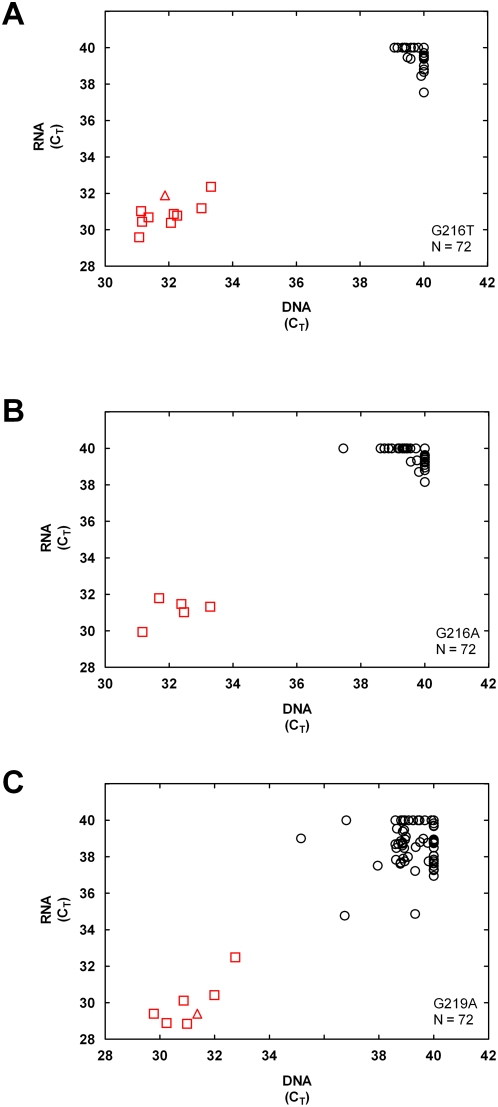
Concordance of mutation assay results in DNA and RNA from 72 FPE tissue specimens for which both RNA and DNA data was available. Circles: (Ο) Samples called wild type by both PCR and DNA sequencing; Squares (□): samples called mutant by both PCR and DNA sequencing; Triangles (▵): samples called mutant by ASB-PCR but wild type by sequencing. A: Kras G216T, assay Mut1.1. B: Kras G216A, assay Mut2.1. C: Kras G219A, assay Mut3.1.

Using ASB-PCR for mutation analysis of RNA has the advantages that only one nucleic acid extraction is needed and the RNA can be processed for gene expression analysis of the same samples. Therefore, we wanted to compare the concordance of results from ASB-PCR analysis of DNA and RNA extracted from the same clinical specimens. RNA was extracted from 72 of the original 82 colorectal cancer FPE tissue specimens described above (the remaining samples having been depleted) and ASB-PCR assays for all Kras mutations were carried out. Figures 5 A–C show scatter plots of RNA vs. DNA C_T_ values for assays Mut1, Mut2 and Mut3, representing three of five mutation assays for which mutation-positive samples were identified. Two distinct clusters were observed for each assay, indicating concordance of mutation assignments in RNA and DNA extracts. Assays Mut4 and Mut5 also produced clusters very similar to those shown in Figure 5 (data not shown).

## Discussion

The ASB-PCR mutation detection method reported here has features that distinguish it from other mutation assays. Principal among these features is the high level of selectivity possible without the need for proprietary reagents other than those normally required for TaqMan® real-time PCR. The assay design rules reported here, combining the use of an oligonucleotide blocker with an allele-specific PCR primer, consistently produced mutation assays with selectivity greater than 1∶1000 which was an improvement over the selectivity achieved using allele-specific primers alone. Moreover, the reagents and workflow process for the ASB-PCR method is compatible with standard protocols for real-time RT-PCR, enabling mutation analysis to be performed alongside gene expression assays of the same samples using a single technology platform. This last point is significant because gene expression profiles generated by RT-PCR have proven to be diagnostically valuable in clinical practice [32].

Combining competitive blockers with allele-specific PCR primers overcomes one of the major limitations of mutation detection that relies on allele-specific primers alone. Several studies have addressed the effect of primer∶template mismatches on the efficiency of extension by *Taq* or other polymerases [18], [27]. The general consensus that has emerged is that purine-pyrimidine (A∶C, C∶A, G∶T, T∶G) and pyrimidine-pyrimidine (T∶C, C∶T, T∶T, C∶C) mismatches extend efficiently whereas purine-purine (A∶G, G∶A, A∶A, G∶G) mismatches do not. Consistent with these earlier reports, our assays Mut1.2 (A∶G mismatch), Mut7.1 and Mut6.1 (both G∶G mismatch), exhibit very high selectivity using only allele-specific primers, as expected for purine-purine mismatches. The primer∶template mismatch of T∶G was found by Lattora et al. [18] to be the most permissive mismatch in terms of *Taq* extension in PCR. Likewise, assays Mut2.2, Mut3.2 and Mut 4.2 (all T∶G mismatch) exhibit very poor selectivities in their unmodified forms and proved the most refractory to improvement from low T_m_ primers. Competitive blocker oligonucleotides of various types have been used by several investigators to suppress amplification of mismatched templates in PCR [14], [15], [33], [34]. We evaluated non-extendable oligonucleotide blockers with the discriminating base located approximately in the center of the sequence in order to maximize the T_m_ difference between matched and mismatched templates [35]. Blockers of this type improved discrimination for most, but not all, of our assays. Low T_m_ primers and blockers used in combination produced the greatest benefit in terms of selectivity (average ΔC_T_ = 13.1) without measurable losses in sensitivity.

Two features of our competitive blocker oligonucleotides contributed to their effectiveness in suppressing amplification of wild type templates: first, the location of the discriminating base in the middle of the blocker sequence; second, the partial overlap with the sequence of the allele-specific primer. Blockers based on this principle preferentially bind perfectly-matched templates and inhibit binding of mismatched primers. We showed that perfectly-matched primers were refractory to inhibition by blockers over a wide T_m_ range. In our studies discrimination was improved when blockers were used in conjunction with low T_m_ primers and, importantly, extension from the permissive T∶G mismatches (assays Mut2.2, Mut 3.2 and Mut 4.2) was further suppressed by at least 3 C_T_s when blockers were added.

Having the option to use either the forward or reverse primer as the allele-specific primer increases the opportunity to develop an optimal ASB-PCR assay. Knowledge of the primer∶template mismatch can aid in selection of the allele-specific primer (forward vs. reverse) as illustrated by our variable results obtained with the two versions of the Kras Mut3 assay. In most cases in the current study, however, strand selection had little impact on the selectivity of the assay, suggesting that the method is robust and independent of sequence context. This is important because the design of ASB-PCR assays is constrained by the need to encompass the sequence surrounding the variant base.

A noteworthy feature of ASB-PCR is the consistently high level of selectivity obtained using the assay design rules described herein. In addition to the Kras mutation assays reported here we have used the ASB-PCR assay design rules to develop assays for 13 different mutations in BRAF, PIK3CA, p53, and CYP2D6. With the exception of an assay for a mutation in BRAF (T600A) all of the assays have selectivity greater than 10 ΔC_T_. The BRAF T600A assay, in which the forward primer is allele-specific, has selectivity of 6.5 ΔC_T_ (A∶A mismatch) whereas the assay in which the reverse primer is allele-specific, has selectivity of 15 ΔC_T_ (T∶T mismatch). Thus, consistently high selectivity mutation assays can be developed using the ASB-PCR design rules without the need for multiple cycles of reagent design and optimization.

The utility of ASB-PCR assays for analysis of nucleic acids extracted from FPE tissues is of particular interest. Previously, we have noted the value of archived FPE specimens for discovery of gene expression profiles that predict clinical outcomes [24], [32], [36]. The ASB-PCR assay method reported here enables mutation analysis in the same archival specimens used for profiling gene expression. Several lines of evidence support the validity of our mutation assays for RNA extracted from FPE tissue specimens. First, the frequency of mutations we observed in colorectal tumors was similar to the frequency determined by sequencing in colon cancer [28]. Second, the results from ASB-PCR assays of nucleic acids extracted from FPE specimens were highly concordant with analysis of the same specimens by conventional Sanger sequencing. Third, analyses of RNA and DNA extracted from the same FPE specimens were highly concordant. It has been widely reported that deamination of cytosine or adenine caused by formalin fixation produces uracil and hypoxanthine residues in their place, respectively, resulting in what appear to be C∶T (G∶A) or A∶G (T∶C) transition mutations [37], [38]. These alterations are indistinguishable from biological mutations with the exception of uracil formation in genomic DNA which can be ablated by pre-treatment with uracil-N-glycosylase [39]. We have also observed a high frequency of randomly distributed C–T transitions, presumably the result of formalin-induced deamination, in resequencing of nucleic acids extracted from FPE specimens (unpublished observations). These transitions are more common in RNA than DNA but were never present at a frequency greater than 2% at any given base position. The high level of concordance of our ASB-PCR results with RNA and DNA extracted from the same FPE specimens is evidence that the mutations we detect are not the result of formalin-induced deamination.

The ability to detect mutations in RNA potentially provides additional information not possible to discern from analysis of DNA. Several investigators have noted that differential expression of alleles is common in the human genome [40]–[42]. Hodgson and coworkers [43] reported that RNA extracted from breast tumors was enriched by as much as 10,000-fold for mutant p53 sequences as compared with DNA extracted from the same specimens. We did not observe a similar enrichment of Kras mutations in RNA as compared with DNA in colorectal tumors, suggesting that generalizations regarding differential expression of mutant alleles are not possible. Further studies are needed to determine if differential expression of mutant alleles, such as those reported for p53, correlate with clinical outcomes.

Finally, it is noteworthy that evidence for the practical clinical utility of the assay methodology described here and applied to FPE tissue RNA has recently been presented. Specifically, the Kras mutation assays described above were used to screen tumor RNA from metastatic colon cancer patients prior to their treatment with the anti-EGFR monoclonal antibody Cetuximab, and have demonstrated a profoundly strong correlation between presence of Kras mutation and failure to respond to this therapeutic agent [44].

## Supporting Information

Table S1 Summary of assay oligonucleotide components. Sequences are listed in 5′-3′ order from left to right. Discriminating primers are underlined with 3′ bases in boldface. Locked Nucleic Acids (LNAs) are represented by capital letters. PO4: 3′-phosphate. Melting temperatures (Tms) were determined using the Primer Express™ software package. The region of Kras against which assays were designed is also shown with codons 12 and 13 listed in boldface. NA: Not Applicable.
(0.06 MB DOC)

Table S2 Mutation calls by ASB-PCR analysis and sequencing
(0.03 MB DOC)

Figure S1 CT response as a function of discriminating primer Tm. Primer Tms were altered by lengthening or shortening from the 5′ end while keeping the 3′ end anchored on the variant site. Primer lengths varied from 13–23 bases. Sixty nanograms cell line RNAs were used as template for assays Mut1-Mut5 and Mut7. Thirty nanograms HeLa DNA (wild type template) or 27,300 copies synthetic DNA (mutant template) were used for assay Mut6. Squares represent the indicated assay applied to mutant template. Circles represent the indicated assay applied to wildtype template. Filled symbols represent assays without blocker added. Open symbols represent assays with 3600 nM blocker. A.→Final Mut1.1 assay: 50°C discriminating primer Tm with blocker. B.→Final Mut2.1 assay: 48.9°C discriminating primer Tm with blocker. C.→Final Mut3.1 assay: 45°C discriminating primer Tm with blocker. D.→Final Mut4.1 assay: 50.6°C discriminating primer Tm with blocker. E.→Final Mut5.1 assay: 50.2°C discriminating primer Tm with blocker. F.→Final Mut6.1 assay: 50.5°C discriminating primer Tm with blocker. G.→Final Mut7.1 assay: 51.4°C discriminating primer Tm with blocker. H.→Final Mut1.2 assay: 49.7°C discriminating primer Tm with blocker. I.→Final Mut2.2 assay: 48.5°C discriminating primer Tm with blocker. J.→Final Mut3.2 assay: 45.3°C discriminating primer Tm with blocker. K.→Final Mut4.2 assay: 50.6°C discriminating primer Tm with blocker.
(0.23 MB DOC)
