# Supplementary material for: Mutation Detection by Real-Time PCR: A Simple, Robust and Highly Selective Method
Source: PLoS One. 2009 Feb 25;4(2):e4584. doi: 10.1371/journal.pone.0004584 (PMC2642996; doi:10.1371/journal.pone.0004584)
Supplement: Table S1 — Summary of assay oligonucleotide components. Sequences are listed in 5′-3′ order from left to right. Discriminating primers are underlined with 3′ bases in boldface. Locked Nucleic Acids (LNAs) are represented by capital letters. PO4: 3′-phosphate. Melting temperatures (Tms) were determined using the Primer Express™ software package. The region of Kras against which assays were designed is also shown with codons 12 and 13 listed in boldface. NA: Not Applicable. (0.06 MB DOC) [file pone.0004584.s001.doc]

Table S1

| **Assay** | **Forward Primer** | **T_m_** | **Probe** | **T_m_** | **Blocker** | **T_m_** | **Reverse Primer** | **T_m_** |
| --- | --- | --- | --- | --- | --- | --- | --- | --- |
| **wildtype1-1** | aaacttgtggtagttggagctg**g** | 60.6 | cacTcTTgcctacgc | 66 | NA |  | tgattctgaattagctgtatcgtcaa | 58.3 |
| **Mut1.1** | ttgtggtagttggagctg**t** | 50.0 | " | " | ttggagctggtggcgtagg-PO4 | 60.8 | " | " |
| **Mut2.1** | tgtggtagttggagctg**a** | 48.9 | " | " | " | " | " | " |
| **Mut4.1** | aacttgtggtagttggagct**a** | 50.6 | " | " | " | " | " | " |
| **Mut5.1** | acttgtggtagttggagct**t** | 50.2 | " | " | " | " | " | " |
| **Mut6.1** | acttgtggtagttggagct**c** | 50.5 | " | " | " | " | " | " |
| **Mut7.1** | tgtggtagttggagctg**c** | 51.4 | " | " | " | " | " | " |
| **wildtype1-2** | tgtggtagttggagctggtg**g** | 59.6 | cactCtTgCctaCg | 65 | NA |  | tgattctgaattagctgtatcgtcaa | 58.3 |
| **Mut3.1** | gtagttggagctggtg**a** | 45 | " | " | gctggtggcgtaggc-PO4 | 52.6 | " | " |
| **wildtype2-1** | cccaggtgcgggagaga | 59.3 | ccacaagtttatattcagtcattttcagcagg | 65.2 | NA |  | gcactcttgcctacgcca**c** | 58.1 |
| **Mut1.2** | " | " | " | " | cctacgccaccagctccaa-PO4 | 60.8 | ctcttgcctacgcca**a** | 49.7 |
| **Mut2.2** | " | " | " | " | " | " | ctcttgcctacgcca**t** | 48.5 |
| **Mut4.2** | " | " | " | " | " | " | ctcttgcctacgccac**t** | 50.6 |
| **wildtype2-2** | cccaggtgcgggagaga | 59.3 | taccacaagtttatattcagtcattttcagcagg | 65.5 | NA |  | caaggcactcttgcctacg**c** | 59.6 |
| **Mut3.2** | " | " | " | " | gcctacgccaccagc-PO4 | 52.6 | gcactcttgcctacg**t** | 45.3 |
|  |  |  |  |  |  |  |  |  |
| **k-ras amplicon region** | CCCAGGTGCGGGAGAGAGGCCTGCTGAAAATGACTGAATATAAACTTGTGGTA GTTGGAGCT**GGTGGC**GTAGGCAAGAGTGCCTTGACGATACAGCTAATTCAGAATCA | | | | | | | |
