# Supplementary material for: Mutation Detection by Real-Time PCR: A Simple, Robust and Highly Selective Method
Source: PLoS One. 2009 Feb 25;4(2):e4584. doi: 10.1371/journal.pone.0004584 (PMC2642996; doi:10.1371/journal.pone.0004584)
Supplement: Table S2 — Mutation calls by ASB-PCR analysis and sequencing (0.03 MB DOC) [file pone.0004584.s002.doc]

**Table S2:**

| Mutation | PCR | Sequencing |
| --- | --- | --- |
| G216T | 8 | 7 |
| G216A | 2 | 2 |
| G219A | 7 | 6 |
| G215A | 0 | 0 |
| G215T | 2 | 2 |
| G215C | 0 | 0 |
| G216C | 0 | 0 |
| Total Mutant Positive | 19 | 17 |
| Total Mutant Negative | 25 | 27 |

N = 44 tumors
