# Supplementary figures and images for: Mutation Detection by Real-Time PCR: A Simple, Robust and Highly Selective Method
Source: PLoS One. 2009 Feb 25;4(2):e4584. doi: 10.1371/journal.pone.0004584 (PMC2642996; doi:10.1371/journal.pone.0004584)

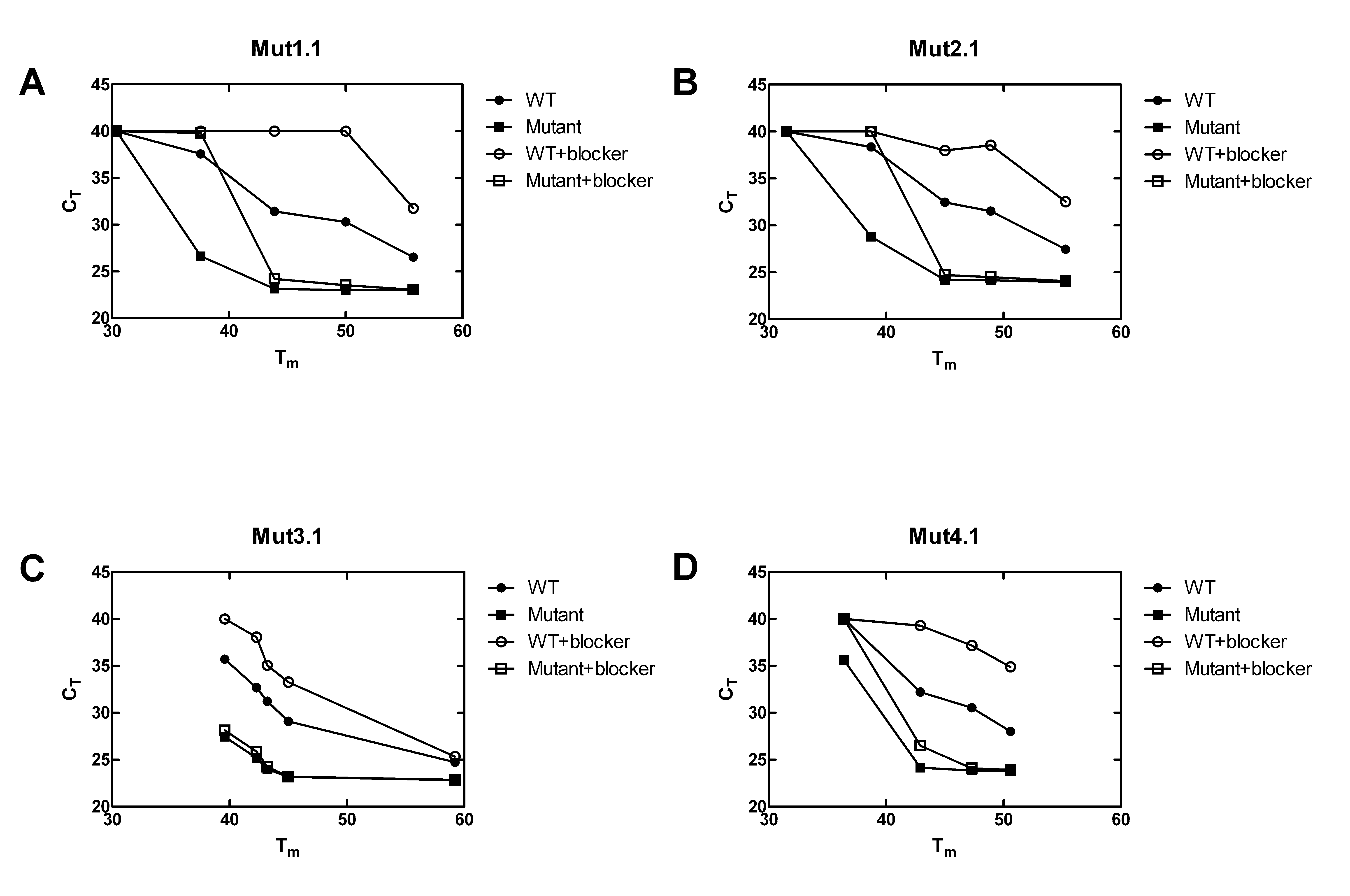


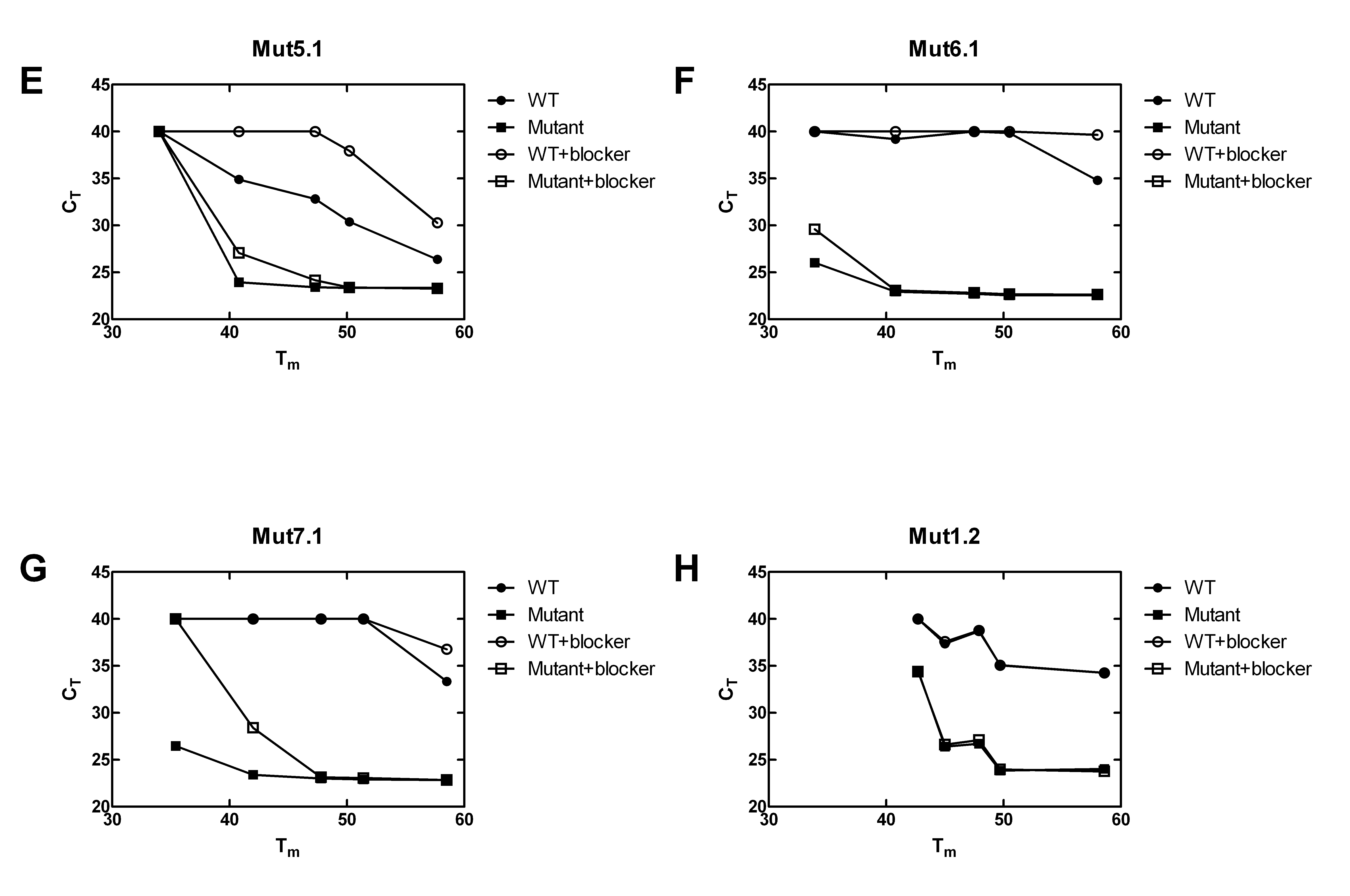


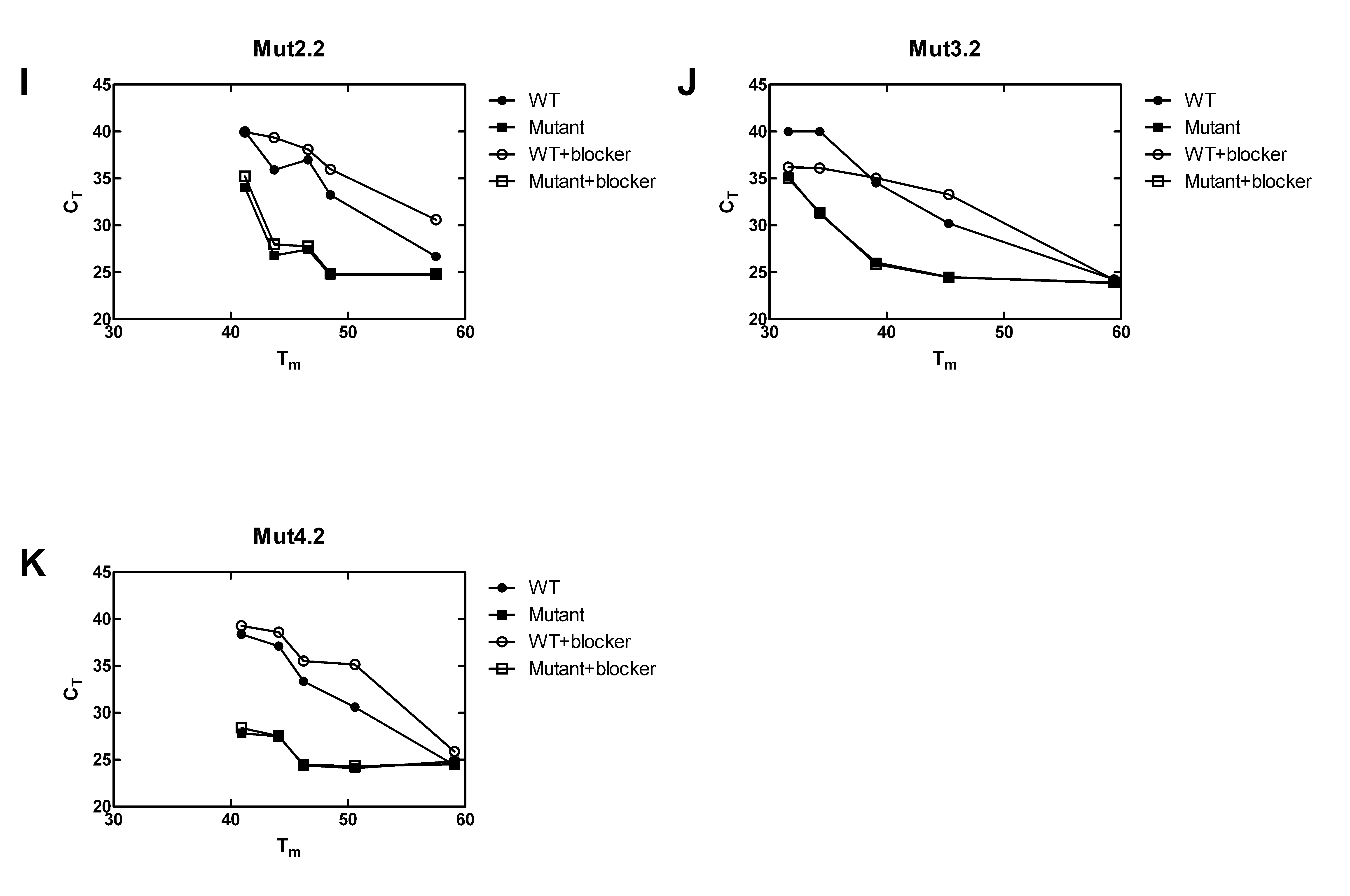

Supplement: Figure S1 — CT response as a function of discriminating primer Tm. Primer Tms were altered by lengthening or shortening from the 5′ end while keeping the 3′ end anchored on the variant site. Primer lengths varied from 13–23 bases. Sixty nanograms cell line RNAs were used as template for assays Mut1-Mut5 and Mut7. Thirty nanograms HeLa DNA (wild type template) or 27,300 copies synthetic DNA (mutant template) were used for assay Mut6. Squares represent the indicated assay applied to mutant template. Circles represent the indicated assay applied to wildtype template. Filled symbols represent assays without blocker added. Open symbols represent assays with 3600 nM blocker. A.→Final Mut1.1 assay: 50°C discriminating primer Tm with blocker. B.→Final Mut2.1 assay: 48.9°C discriminating primer Tm with blocker. C.→Final Mut3.1 assay: 45°C discriminating primer Tm with blocker. D.→Final Mut4.1 assay: 50.6°C discriminating primer Tm with blocker. E.→Final Mut5.1 assay: 50.2°C discriminating primer Tm with blocker. F.→Final Mut6.1 assay: 50.5°C discriminating primer Tm with blocker. G.→Final Mut7.1 assay: 51.4°C discriminating primer Tm with blocker. H.→Final Mut1.2 assay: 49.7°C discriminating primer Tm with blocker. I.→Final Mut2.2 assay: 48.5°C discriminating primer Tm with blocker. J.→Final Mut3.2 assay: 45.3°C discriminating primer Tm with blocker. K.→Final Mut4.2 assay: 50.6°C discriminating primer Tm with blocker. (0.23 MB DOC) [file pone.0004584.s003.doc]
